# Markets in crypto-assets regulation: Does it provide legal certainty and increase adoption of crypto-assets?

**DOI:** 10.1186/s40854-022-00432-8

**Published:** 2023-01-10

**Authors:** Tina van der Linden, Tina Shirazi

**Affiliations:** 1 Faculty of Law, Transnational Legal Studies, Vrije Universiteit Amsterdam, 1081 HV Amsterdam, The Netherlands; 2 Zurich, Switzerland

**Keywords:** MiCA regulation, Crypto-assets, Legal certainty, Blockchain, Distributed ledger technology, Utility tokens, Stablecoins, Asset-referenced tokens, e-money tokens

## Abstract

This study discusses the European Union’s proposal for a Regulation on Markets in Crypto-Assets, now subject to formal approval by the European Parliament. The objective is to explore whether it will positively impact the adoption of crypto-assets in the financial sector. The use of crypto-assets is growing. However, some stakeholders in the financial service sector remain skeptical and hesitant to adopt assets that are yet to be defined and have an unclear legal status. This regulatory uncertainty has been identified as the primary reason for the reluctant adoption. The proposed regulation (part of the EU’s Digital Finance Strategy) aims to provide this legal certainty for currently unregulated crypto-assets. This study investigates whether or not the proposed regulation can be expected to have the intended effect by reviewing the proposed regulation itself, the opinions and reactions of the various stakeholders, and secondary literature. Findings reveal that such regulation will most likely not accelerate the adoption of crypto-assets in the EU financial services sector, at least not sufficiently or as intended. Some suggestions are made to improve the proposal.

## Introduction

According to Bill Gates, “Banking is necessary; banks are not” (Amberber 2015).

### Setting the stage

Crypto-assets, specifically Bitcoin, date back to 2009 and the global financial crisis, which resulted in significant drops in real gross domestic product (GDP) and corporate profits and a decline in trust in the financial sector (Huibers 2019). Following the crash, the very nature of banking was scrutinized, particularly regarding its function and role in society. Moreover, whether Bitcoin’s launch was a coincidental event or a deliberate strategic decision (Nakamoto 2009), it undoubtedly transformed the potential for decentralized systems in the financial sector. Bitcoin is a peer-to-peer electronic cash system that enables money in this cryptocurrency to be sent online, pseudonymously, and directly to a recipient without using a centralized intermediary, such as a bank.

Bitcoin is the first application of this distributed ledger technology (DLT), which can be used for cryptocurrencies and other purposes. Cryptocurrencies are thus a subset of the broader category of crypto-assets and can represent various products and be used for various purposes. The European Central Bank (ECB) has described crypto-assets as a new phenomenon that may increase financial system risks (Chimienti et al. 2019). Article 3(2) of the proposed Regulation on Markets in Crypto-Assets (MiCA Regulation)^1^ defines crypto-assets as a digital representation of value or rights that can be transferred and stored electronically using DLT or similar technology.

Crypto-assets are increasingly being integrated into financial services applications to enable near real-time transactions, accurate data recording, and more efficient payment processes. However, one of the primary barriers to implementing these assets is a lack of legal certainty (Dutch Banking Association 2020).

Crypto-assets have emerged as a major trend in digital transformation. Indeed, the World Economic Forum estimates that up to 10% of global GDP will be stored and transacted via DLT by 2027 (Tummala 2020), with the tokenized market having the potential to reach $24 trillion by that year (Fidelity Digital Assets 2020). In a survey conducted by Fidelity Digital Assets among nearly 800 institutional investors in the United States and Europe from November 2019 to March 2020, 36% of the respondents stated that they were currently investing in digital assets (Fidelity Digital Assets 2020). Although crypto-assets are being used in various industries, their adoption is particularly evident in financial services, where the OECD has identified a significant increase in the number of potential applications, including tokenized equities, bonds, and commodities (OECD 2021). The COVID-19 pandemic, which forced banks to limit face-to-face interactions and adapt quickly, may have boosted this. Indeed, according to Boston Consulting Group, a new wave of digital disruption in banking is inevitable (Erlebach 2020).

In recent years, the financial services sector has faced numerous challenges, particularly due to the financial crisis, which required banks to work hard to regain society’s trust. Using new technologies is recognized as one method of improving financial operations (Kou et al. 2021). The European Parliament has identified that, if properly regulated, crypto-assets can result in huge efficiency gains in the financial sector by transforming the functioning of financial markets to facilitate the exchange of value without the need for a central authority or intermediary (Saulnier 2020, 3). It is estimated that blockchain technology could reduce infrastructure costs by 30% for the top ten banks alone, resulting in savings of $8 to $12 billion (Casey 2018, 5). The financial services sector is the world’s largest user of ICT, and according to a Policy Department study requested by the European Parliament’s Committee on Economic and Monetary Affairs (ECON), over 5,100 types of crypto-assets existed in 2020 (Houben et al. 2020, 25). However, although the market capitalization of crypto-assets rose to $2 trillion in May 2021, it fell to $1.3 trillion in August 2021, highlighting both the market’s potential and fragility (Statistica 2022).

Areas that can benefit from using DLT include payments, digital identity/Know Your Customer (KYC), securities settlement, and trade finance. The Dutch Banking Association (2020, p. 4) stated that crypto-assets can create opportunities for the financial sector, particularly in the areas of issuance, trading and especially custody services. Subsequently, it identified high-performing crypto exchanges and traditional finance market operators as the two types of players with the greatest potential to achieve significant market shares in the crypto-asset markets (Dutch Banking Association 2020, p. 16). Therefore, this paper will focus on the MiCA Regulation’s implications for both traditional financial market operators (e.g., banks) and smaller players (e.g., fintechs). Traditional financial market operators are important to consider because they are large players who can significantly impact the world’s financial infrastructure, impact average consumer demands, and potentially seize market capitalization through their role as facilitators. Meanwhile, fintech companies have grown, with crypto-assets providing new opportunities for these smaller companies, which include parties that use emerging technology to automate and improve financial service processes. Because the proposed MiCA Regulation will affect a wide range of players, it will be interesting to explore how the regulation will play out in different markets and whether it will explore or discourage crypto-asset use.

However, regulation must catch up and provide legal certainty for the financial sector to make greater use of crypto-assets. Having acknowledged the urgent need for appropriate regulation, and the importance of this for the adoption of crypto-assets, the European Commission then proposed the MiCA Regulation.

### Research question

This study discusses whether the proposed MiCA Regulation will create legal certainty, allowing for the increased adoption of crypto-assets in financial services.

### Methodology

Through a review of the literature, this study adopts a primarily legal-sociological approach to examining legislation in the context of larger society. Legal and academic papers, opinions, blog posts, and other materials were analyzed for qualitative and dogmatic research. We also considered a doctrinal aspect by using the proposed regulation (a primary source of law) as a benchmark for comparing opinions and expected societal effects. Given that the regulation was only proposed in 2020 and has yet to enter into force, we will rely heavily on recent sources. Similarly, crypto-assets have only recently emerged and become subject to legislation; therefore, the available academic literature is relatively limited.

Because the proposed MiCA Regulation is the focus of this paper’s research, we will not examine the phenomenon of decentralized finance (DeFi) (Maia and Vieira dos Santos 2021).

The remainder of this paper is structured as follows: “Background of crypto‑assets” section defines crypto-assets and explains why their greater adoption is desirable. “Research question” provides an overview of current crypto-asset legislation and the context of the MiCA Regulation to identify what is uncertain in the current legal framework. “Proposed MiCA regulation” section examines the proposed regulation, its goals, and claims to provide legal certainty. “Impact of MiCA on crypto‑asset adoption” section contains a critical analysis of the proposal based on stakeholder feedback. Finally, “Conclusion” section concludes the paper. The conclusion provides an answer to the research question.

## Background of crypto-assets

### Crypto-assets explained

We start with the concept of a token. Tokens are legal instantiations of a share of an asset, a set of permissions, or claims held by the token’s bearer (Global Digital Finance 2018). Examples of traditional physical tokens include plastic coins giving access to a fairground ride or insertable into a slot machine, bingo cards, and traditional bearer bonds and checks. We also use digital tokens these days, such as QR codes, discount codes, and permissions in digital form. Crypto-assets are digital tokens created with cryptography and DLT that are neither issued nor guaranteed by a central bank or public authority and can be used as a means of exchange, investment, or access to goods or services. In other words, crypto-assets can be viewed as a digitally transferable representation of a value or a right. They differ from traditional assets in that the cryptographically secured transactions that create and track crypto-assets are stored on a distributed ledger.

The following is a brief note on terminology: blockchain is a type of distributed ledger that consists of interlocking hashed blocks of transaction data, forming a chain. The method by which the peer-to-peer network members agree on the link between the blocks (i.e., the consensus mechanism) can vary and is not yet stable. To avoid creating terminological issues and confusion, and to be as technology-neutral as possible, we have chosen to use the term distributed ledger rather than blockchain in this paper and the proposed MiCA Regulation. For an overview of academic research on blockchain in business and economics, see Xu et al. (2019).

Users are not required to identify themselves to participate in the peer-to-peer network on which a distributed ledger is stored; this is heavily dependent on how a network is organized. It may be sufficient to simply download and install the network’s software to become a participant, in which case it may be difficult to determine who a new participant is or where they are located. Participants may, therefore, operate strictly under pseudonyms, which may amount to anonymity for practical purposes.

Transactional data stored on a distributed ledger cannot be changed or deleted normally. Of course, another transaction can undo the effects of an earlier transaction (e.g., recrediting an amount that was previously debited); however, the original transaction cannot be changed or deleted. This feature, known as immutability, allows network participants to trust the data stored on the ledger.

Crypto-asset owners typically keep these assets in their wallets. Transfers are executed by using asymmetric encryption, a clever mechanism using a key pair consisting of a private and a corresponding public key. A message encrypted by a private key (in this case, initiating a transaction) can only be decrypted by the corresponding public key. However, even if they are obviously mathematically related, the private key can never be deduced from the public key. Thus, a private key serves as a signature, whereas the corresponding public key serves as an address. A wallet can be considered a private key (or key storage) designated to the outside world by the corresponding public key. All network peers can be confident that a transaction signed with a private key comes from the wallet indicated by the corresponding public key, which allows the message to be decrypted. The transaction history stored on the ledger is used to calculate a person’s wallet balance. Thus, crypto-assets are not kept in the wallet like physical coins or other tokens kept in a physical wallet.

Trading on a distributed ledger does not always require humans to sit at computers and type commands. Instead, the distributed ledger enables something akin to vending machines: computer-operated wallets can automatically handle any transactions sent to them by human users or other computer programs. These programs are referred to as smart contracts. Several related smart contracts form what is known as a distributed autonomous organization.

### Types of crypto-assets

In general, three types of crypto-assets can be distinguished based on their intended use. However, their distinctions are not always clear, and mixed-type crypto-assets exist. Note that creating crypto-assets is very accessible and easy. Alongside serious, professional business initiatives, there are semi-serious initiatives, trial balloons, things that begin as a joke but have the potential to become more serious, and pure jokes and fun experiments that begin simply because an opportunity presents itself.^2^ Moreover, it is not always easy for investors, particularly non-professional investors, to differentiate between them–another reason for regulation.

*Cryptocurrencies*, the original application of this type of DLT, are one type of crypto-assets. To name a few, Bitcoin, Ether, Binance Coin, Cardano, Polkadot, and Dogecoin are among the most well-known. However, there are numerous others (CoinMarketCap 2022). Cryptocurrencies were created to provide an alternative to traditional fiat currencies issued by governments and enable fast, secure, and anonymous internet payments. They can also serve people who do not have access to a bank account because no intermediaries are required besides the network itself. The exchange rate between a cryptocurrency and a fiat currency is determined by supply and demand, making it highly volatile and unpredictable.

Cryptocurrencies can function as a means of exchange and are therefore sometimes referred to as exchange tokens (Cryptoassets Taskforce 2018). They can potentially be disruptive to the financial sector (Sebastião et al. 2021; Fang et al. 2022). In response to retail banking clients’ and institutional investors’ interest in cryptocurrencies in recent years (Wintermeyer 2021), financial institutions with a high-risk appetite are now offering these currencies to customers, and large institutions have begun investing in them on their own balance sheets. However, while cryptocurrencies have been prevalent among wealthy customers, the risks involved (including the risks associated with issuing cryptocurrencies) have hampered their institutional adoption. The literature has noted that the nature of cryptocurrencies makes it difficult to separate financial and technological risks (Dumas et al. 2021).

The exchange rate between a cryptocurrency and a fiat currency is determined by supply and demand, making it highly volatile and unpredictable (Woebbeking 2020). This makes “investing” in cryptocurrencies a risky endeavor, giving rise to the desire for regulation to protect consumers from misleading advertisements and scams.

Other crypto-assets are commonly referred to as tokens. Tokenization refers to dividing the exclusive right to something into tokens (similar to shares) that can be traded on a distributed ledger. Thus, a startup business plan, or even physical items such as real estate, can be tokenized to produce utility or security tokens, as explained further below. These can be sold to anyone willing to invest in an initial coin offering (ICO) or security token offering (STO) (Spaeth and Peráček 2022). The specific offering and terms are usually explained in the accompanying white paper.

A second type of crypto-assets, *utility tokens*, are defined in MiCA as a type of crypto-asset intended to provide digital access to a good or service available on DLT and only accepted by the token’s issuer, in other words, the DLT equivalent of the fairground coin. Examples include tickets for events (EventMB Studio Team 2021), the Basic Attention Token issued for viewing ads on the Brave browser (Basic Attention Token 2022) and the Golem Network Token used on the Golem marketplace for computing power (Golem Network 2022). As demonstrated by these examples, a utility token can be purchased as a ticket to access goods or services, acquired in an ICO as a byproduct of an investment or an expression of support, or used as a means of exchange on a platform dedicated to a specific type of activity. Thus, utility tokens enable creativity.

Access to goods via a utility token rather than fiat currency or traditional investment is a step toward the token economy (Voshmgir 2021). The transition from a centralized platform economy to a decentralized token economy may provide opportunities for the financial sector such as increased liquidity, transparency, and real-time transactions. However, the challenges of ensuring appropriate legislative and regulatory frameworks currently limit financial institutions’ ability to reap these benefits.

A third type of crypto-assets, *security tokens*, represent an investment. Like traditional securities, they provide rights in the form of ownership rights or entitlements. They may also be regulated, just like traditional securities. In the United States, for example, whether or not they are regulated is determined by the Howey test^3^: if they involve money being invested in a common enterprise, where profit is expected solely from the promoter’s or a third party’s efforts, the asset constitutes security and, as such, is regulated (including registration with the Securities and Exchange Commission). In contrast, the regulatory landscape for security tokens in Europe is fragmented and unclear (Burilov 2019), which is yet another reason for the proposed MiCA Regulation.

Using security tokens instead of traditional securities may provide many advantages. For starters, the decentralized nature of DLT technology may improve operational efficiency by allowing transactions to be completed more quickly and cheaply. As disintermediation increases, DLT may reduce paperwork, simplify reporting processes, and reduce issuance fees. Second, asset fractionalization allows investors to buy tokens representing a very small percentage of the underlying assets, increasing accessibility for a broader range of investors and thus unlocking capital and market liquidity. Finally, unique DLT characteristics such as immutability, which allows data to be tamper-proof, will increase transparency. Security tokens on DLT can easily be traced through their provenance, and transaction histories can be verified.

However, these tokens pose certain challenges and raise questions. Because their legal status remains unclear, what exactly do we get (in legal terms) when purchasing a token? Is it legal to fractionalize real estate ownership? Who are the parties involved in an STO? Can security token issuers tell whether they are dealing with consumers (and thus subject to consumer protection legislation) or professionals? What about anti-money laundering (AML), KYC, and counter-terrorism financing (CTF) regulations? Are they applicable? Who is in charge of ensuring compliance? Overall, there are still many questions to be answered and a great deal of legal uncertainty to be resolved.

Then, to add to the complication, hybrid tokens combine one or more of the previously mentioned functions. Security tokens may include some utility, utility tokens may also be used for investment, cryptocurrencies may be used for speculation, and so on. In contrast to MiCA, which is the subject of this paper, the Liechtenstein Blockchain Act is based on a “Token Container Model” (Sandner, Nägele, and Gross 2019). A token is viewed as a container that can contain a variety of rights and can also be empty. As a result, there is no need to distinguish between different types of tokens.

Investors must be able to manage the financial risks they take, such as maintaining a certain level of liquidity, diversifying their investment portfolios, and avoiding asset bubbles. The uncertain legal status of crypto-assets is also a risk. Regulatory clarity is thus critical if the EU is to lead the way in allowing crypto-related projects to be launched and implemented. Simultaneously, the various types of crypto-assets have significant potential for the financial sector, emphasizing the importance of the proposed MiCA Regulation for adopting these assets.

## Crypto-assets and the law

This section examines the current legislative framework on crypto-assets to see if there are any gaps that MiCA can explore, the proposed Digital Finance Package, of which MiCA is a component, and MiCA’s objectives. Finally, the issue of definitions and the need for regulation will be examined.

### Background of current legislation on crypto-assets

Various directives and regulation currently govern financial markets in the EU. However, current legislation primarily governs tokens that involve an intermediary (i.e., centralized tokens) rather than fully decentralized tokens. Therefore, although the MiCA proposal was triggered for various reasons, including the EU’s current lack of adequate regulation on crypto-assets, current legislation will remain relevant because the MiCA Regulation will apply only to crypto-assets that are currently unregulated, as shown in Table 1. The European Council recently published the agreed-upon text of MiCA,^4^ and the next step is formal approval by the European Parliament.

**Table 1:** Current legislation on crypto-assets

	Relevant for crypto-assets qualifying as or involving	Aims
MiFID II	Financial instruments	Market transparency and investor protection
AMLD2	Virtual currencies	Prevention of terrorist financing and money laundering
EMD2	Electronic money	Stimulation of electronic money
PSD2	Payment transactions	Stimulation of innovation and competition in payments market

#### MiFID II

The Markets in Financial Instruments Directive framework (MiFID II) consists of a directive (MiFID II)^5^ and a regulation (MiFIR).^6^ When MiFID first went into effect in 2007, it was proposed to be extended to cover more aspects of the investment sphere (Sumsub 2019). However, as the market grew and new financial technologies emerged, the EU amended the directive to create MiFID II while also introducing MiFIR to fill gaps in the previous legislation. MiFIR, which focuses on trading and transaction reporting and transparency, thus supplements MiFID II. MiFIR, as a regulation, is directly applicable in all Member States and thus harmonizes the rules, whereas MiFID II had to be implemented individually in each Member State. The term MiFID II/MiFIR refers to the framework of both MiFID II and MiFIR in this paper.

The MiFID II/MiFIR framework went into effect on January 3, 2018, to increase transparency in European financial markets and strengthen investor protection (AFM 2022). MiFID II/MiFIR governs all crypto-assets considered financial instruments in Article 4(15) MiFID II, which include transferable securities, money-market instruments, units in collective investments, and various derivative instruments (options, futures, swaps, forward rate agreements, and other derivative contracts relating to securities, currencies, commodities in cash or that can be physically settled, derivative instruments for transfer of credit risk or financial contracts for differences) and, more recently, emission allowances.^7^ However, the European Securities and Markets Authority (ESMA) notes that this leaves many crypto-assets unregulated and exposes consumers to substantial risks (ESMA 2019).

Article 4(1)(44) MiFID II defines transferable securities as those classes of securities that are negotiable on the capital market (except for payment instruments), such as:

- Shares in companies and other securities equivalent to shares in companies, partnerships, or other entities, and depositary receipts for shares;
- Bonds or other types of securitized debt, including depositary receipts for such securities;
- Any other securities that give the right to acquire or sell any such transferable securities or give rise to a cash settlement based on transferable securities, currencies, interest rates or yields, commodities, or other indices or measures.

This definition of transferable securities is based on the clear assumption that the security is transferable and negotiable (Peráček 2021). If a security can be traded on the market, it is negotiable. To qualify as a transferable security, an instrument does not have to be traded on the capital market. However, because this definition excludes instruments of payment, it raises concerns and uncertainty about cryptocurrencies, as well as utility tokens and security tokens, because all three types of crypto-assets could be considered instruments of payment and thus unregulated under MiFID II.

Opinions by National Competent Authorities (NCAs) such as Germany’s BaFin (BaFin 2018), the Dutch AFM (AFM 2021), and the Swiss FINMA (Eidgenössische Finanzmarktaufsicht FINMA 2018), ESMA (ESMA 2017), and the ESMA Group of Experts and the Securities and Markets Stakeholder Group (SMSG ESMA) (Securities and Markets Stakeholder Group 2018) have all stated that crypto-assets may qualify as financial instruments, and specifically as transferable securities, if they have the relevant characteristics (Hobza 2020). However, there is no consensus on this issue, leading to regulatory uncertainty and the lack of crypto-asset adoption.

#### AMLD5

Another important piece of legislation to consider is the 5^th^ Anti-Money Laundering Directive (AMLD5),^8^ as published on June 19, 2018, which must be implemented in all EU Member States by January 10, 2020. (Sygna 2020). The goal of this directive is to prevent terrorist financing and money laundering. Although a new version, the AMLD6 (European Commission 2015), has since been proposed, the AMLD5 will be used for this paper because it was the first update to include requirements for cryptocurrencies—and the AMLD6 is still a proposal. AMLD5 governs virtual asset service providers, such as exchanges and custodians, and regulates crypto-assets in the EU. Article 1(2)(d)(18) defines virtual currencies as a digital representation of value that is not issued or guaranteed by a central bank or a public authority, is not necessarily attached to a legally established currency, and does not have the legal status of currency or money, but is accepted as a means of exchange by natural or legal persons and can be transferred, stored, and traded electronically—in other words, including crypto-assets. However, because AMLD5 is a directive rather than a regulation, the interpretation of such crypto-assets is at the discretion of individual Member States, which may contribute to an increased lack of harmonization and, as a result, legal uncertainty within the EU.

The inclusion of the aforementioned definitions in the updated AMLD5 was prompted by an increase in criminal activity involving crypto-assets. Recital 9 notes that the anonymity of virtual currencies allows potential misuse for criminal purposes. Additional governance of exchange services for virtual currencies and custodian wallet providers has therefore been introduced to improve the opportunities for identifying and acting on suspicious activity.^9^ The European Commission considers exchange services between virtual and fiat currencies, as well as custodian wallet providers, to be obliged entities (Sygna 2020), which means that these cryptocurrency exchanges qualify as financial institutions and are thus subject to the same AML/CFT requirements as traditional institutions.

The amended AMLD5 does not apply to crypto-to-crypto exchanges, but it does apply to fiat-to-crypto exchanges. The latter exchanges are governed by FATF Recommendation 16 (crypto travel rule), which requires virtual asset service providers (VASPs) to obtain accurate original and beneficiary information in order to comply with KYC and AML requirements (Sygna 2019). VASPs are a method of transferring virtual assets from one address to another on behalf of a natural or legal person (Sygna 2019). However, while this demonstrates the existence of legislation governing crypto-assets and, in particular, service providers, the legislation takes the form of a directive, which is not directly enforceable. Because Member States and FATF member countries can interpret the directive in their own way, the legislation in the various states is not uniform. In a study by the European Parliament, a side-by-side comparison of the latest FATF standards on virtual assets with the AML/CFT legislation for virtual currencies in AMLD5 found existing legislation on virtual currencies in AMLD5 to be lagging behind what is regarded as the current international AML/CFT standard for crypto-assets (Houben et al. 2020).

#### EMD2 and PSD2

The Second Electronic Money Directive (EMD2)^10^ and the Second Payment Services Directive (PSD2) may also apply to crypto-assets.^11^ However, the wording in these directives and the definitions provided do not exactly contribute to a better understanding of how to qualify specific crypto-assets. A crypto-asset meets the definition of electronic money under Article 2(2) EMD2 only if it is electronically stored, has monetary value, represents a claim on the issuer, is issued upon receipt of funds, is issued to make payment transactions as defined in PSD2, and is accepted by persons other than the issuer (EBA 2019). A payment transaction is defined as placing, transferring, or withdrawing funds in Article 4(5) of PSD2. Funds are defined in Article 4(15) PSD2 as banknotes and coins, scriptural money, and electronic money as defined in Directive 2000/46/EC Article 1(3)(b), which was repealed by EMD2 but contained a similar definition of electronic money. As a result, we have a circular definition that is both broad and ambiguous: according to the Bureau Européen des Unions de Consommateurs (BEUC 2020), any debit card linked to a traditional bank could be considered e-money. This definition has created uncertainty, as evidenced by banks’ request to the European Banking Authority (EBA) in January 2020 to clarify the definition of e-money (BEUC 2020). Stablecoins (to be discussed further in the following section) appear to be excluded from the definition of e-money because, by definition, they are electronic monetary units (payment tokens) that can only circulate from one electronic device to another; something that is not reflected in the current definition (placing, transferring, or withdrawing funds) in Article 4(5) PSD2).

The MiFID II and AMLD5 can be seen as the most impactful and relevant legislation on crypto-assets until the MiCA Regulation enters into force. However, the current scope of EU legislation does not adequately cover crypto-assets, and the definitions provided are currently too broad and unclear. Other legislation affecting crypto-assets includes the EMD2 and the PSD2, both of which were not designed to cover crypto-assets and thus add to legal uncertainty. The Prospectus Directive, which harmonizes requirements for drafting, approving, and distributing prospectuses for offering securities to the public or admitting securities to trading on a regulated market (ESMA 2021), and the Transparency Directive, which requires issuers of securities to meet various obligations, are also noteworthy pieces of legislation (AFM 2016). However, for this paper, these will be disregarded.

### Digital finance package

The European Commission adopted the Digital Finance Package on September 24, 2020, to prepare the EU for the digital age (European Commission 2020). This package includes the digital finance strategy, retail payments strategy, crypto-asset legislative proposals, and digital operational resilience legislative proposals. The MiCA Regulation applies to crypto-assets that are currently unregulated, and the Pilot regime, which is relevant to market infrastructures based on DLT and applies to crypto-assets that are already covered by existing regulations, are among the legislative proposals for crypto-assets. Legislators are now reviewing the various proposals. When the MiCA Regulation goes into effect, Member States and relevant regulators will have eighteen months to implement the system. However, this period does not apply to issuers of e-money and asset-reference tokens, who must comply with the MiCA Regulation as soon as it becomes effective.^12^

The European Commission’s Digital Finance Package has four main goals:^13^

1. Reduced fragmentation in the Digital Single Market for Financial Services, allowing consumers better cross-border access to financial products and allowing fintech start-ups to grow.
2. Adoption of an EU regulatory framework that promotes digital innovation for the benefit of consumers and market efficiency: As stated in “Background of current legislation on crypto-assets” subsection, the current EU legislative framework does not adequately govern the crypto-asset ecosystem. Regulation will provide legal certainty, increasing consumer and investor trust in digital innovation.
3. Establishment of a European financial data space to promote data-driven innovation such as data sharing and open finance, while adhering to the GDPR’s data protection standards.
4. Addressing new challenges and risks associated with digital transformation: new innovative practices bring with them new challenges and risks, which must be addressed. Crypto-assets are prime examples of novel solutions that also pose new risks.

The Digital Finance Package is therefore clearly required on a European level, with the European Commission’s ambitions reflecting the goal of a technology-neutral ecosystem that allows innovation to flourish.

### Defining the problem and the emergence of the MiCA Regulation

#### Need for regulation

The announcement of the introduction of Diem (formerly known as Libra, Read and Schäfer 2020; Handagama 2021), a global stablecoin project initiated by Facebook in June 2019, was arguably the primary reason for the emergence of a harmonized framework for crypto-assets. Although this stablecoin has yet to be launched (and may even be canceled entirely), it has served as a wake-up call for regulators by showing the potential and attractiveness of crypto-assets. Concerns were raised within the European Commission following its announcement that stablecoins could be issued and controlled by the private sector. As a result, the Commission decided to act because Diem has the potential to disrupt the traditional payments market by offering low-fee, scalable, and fast settlement, and because the large number of Facebook users (more than 2 billion) suggests that a commercially issued digital currency could jeopardize financial stability as well as monetary and economic sovereignty (European Central Bank 2020). Fabio Pannetta refers to it as an unacceptably low offer. The payment system is so important to society’s functioning that it must be managed and controlled by a public organization; it cannot be in private hands. This is why central banks exist (Pannetta 2021). A fully harmonized, comprehensive, and binding legal framework is required to prevent regulatory loopholes that would allow the launch of commercially issued digital currencies such as Diem.

Stakeholders’ perspectives raised awareness that using DLT and crypto-assets entailed risks and had various negative consequences. The Financial Stability Board (FSB) pointed out that the increased use of decentralized financial technologies may have implications for the effectiveness and enforceability of current regulatory frameworks, particularly where supervisory mandates focus on the presence of financial intermediaries (Financial Stability Board 2019, 7). Furthermore, it emphasized that decentralized financial technologies could be used to avoid regulation through anonymization, while also raising concerns about regulatory enforcement and increasing jurisdictional uncertainty because of these technologies’ operations regardless of borders (Financial Stability Board 2019, 9).

This awareness prompted the EU Commission to direct the European Supervisory Authorities: EBA, the European Insurance and Occupational Pensions Authority and ESMA to examine the applicability of EU financial law to crypto-assets. The EBA’s 2019*Report with advice for the European Commission* noted that crypto-asset-related activity in the EU is still limited and thus does not yet pose a risk to financial stability (EBA 2019, 29). However, it was also stated that some crypto-assets not covered by current EU financial services legislation are extremely risky in terms of consumer protection, operational resilience, and market integrity. Meanwhile, the crypto-asset ecosystem has grown significantly in importance and size since 2019, making the need to address risks even more pressing.

Although ESMA’s *Advice: Initial Coin Offerings and Crypto-assets* issued in 2019 found that the crypto-asset market’s small size meant that financial stability issues had not yet been observed, ESMA was concerned about risks to investor protection (ESMA 2019, 39). The most serious of these threats are fraud, cyber-attacks, money laundering, and market manipulation. ESMA also stated that only a small percentage of crypto-assets qualify as MiFID financial instruments and electronic money; thus, a large percentage of crypto-assets are not covered by financial services legislation. ESMA identified many risks associated with crypto-assets. First, regulators have identified risks in asset issuance, distribution, and storage and risks specific to DLT (ESMA 2019, 13). Second, it must be determined whether crypto-asset platforms have the resources required to effectively address risks, whether these platforms establish and maintain adequate arrangements and procedures, whether adequate measures are in place to prevent potential conflicts of interest, and whether these platforms provide equal access to services (ESMA 2019, 14). Finally, DLT technology may pose risks related to the technology itself, governance, territoriality, the role of miners in transaction verification, privacy concerns, and the possibility of fraud or illicit activities (ESMA 2019, 16).

The European Commission also conducted a *Study on Blockchains*, in which legal certainty and regulatory clarity were identified as key catalysts for blockchain development while also being identified as key barriers to adoption (European Commission 2018, 8). Concerns about legal compliance and liability, potential barriers to unleashing blockchain’s socioeconomic potential, the need to protect fundamental legal principles, and the legal issue of tensions between blockchain and legal reality are among the legal issues identified in the research. The legal reality refers to situations in which legal ownership of an asset changes but this is not reflected in the chain (European Commission 2018, 8). The EU then took preliminary steps to incorporate crypto-assets into the scope of EU legislation by amending the AMLD5, which only covers a subset of the crypto-asset uses identified. The EU Member States were required to act by early 2020 and incorporate AMLD5 into their national legislation. However, the implementation of this directive clearly suffered from a lack of uniform interpretation, with each Member State adopting a different set of rules, leading to greater fragmentation of rules rather than increased harmonization. Furthermore, ALMD5’s definition of virtual currencies was insufficient to cover the full range of crypto-assets.

“Background of current legislation on crypto-assets” section identifies some gaps in the current legislative framework’s application to crypto-assets. For example, MiFID poses difficulties for Member States NCAs in defining the term financial instrument (ESMA 2019, 5). The fact that the definition in Article 4(15) MiFID II leaves room for interpretation is particularly problematic because Member States do not necessarily interpret directives uniformly. ESMA therefore stated that the lack of applicable financial rules exposes consumers to significant risks (ESMA 2019, 5). Meanwhile, NCAs pointed out that existing regulations were not written with crypto-assets in mind, while also acknowledging that classifying crypto-assets as financial instruments could give them unwanted legitimacy. The necessary supervisory tools may not yet be in place (ESMA 2019, 21). A new regulation provides greater clarity on the types of services that crypto-asset providers offer, particularly custody and safekeeping services, settlement concepts, and DLT-specific risks (ESMA 2019, 36). As a first step toward greater market clarity, the European Commission developed a more detailed taxonomy for the classification of crypto-assets.

#### Emergence of MiCA regulation

Stablecoins such as asset-referenced tokens and e-money tokens are the primary target of the proposed MiCA Regulation because they are currently outside the scope of financial services legislation. The European Commission considered two options for dealing with stablecoins: the first was to create a bespoke legislative regime to address the risks posed by stablecoins, and the second was to regulate stablecoins under the Electronic Money Directive.

The first option, a bespoke legislative regime for stablecoins, would address financial stability vulnerabilities while imposing specific disclosure requirements on stablecoin issuers and reserve backing the stablecoin. Disclosure requirements are important because they require all relevant information to be made available to the public as soon as possible. Imposing transparency and trust requirements on stablecoins would increase transparency and trust, allowing for greater adoption in the financial sector. Full reserve backing is important for stablecoins because it requires issuers to keep 100 percent guaranteed reserves against the currency reported as reserve-backed (Weber 2019). This will build trust in the fact that a fully-backed stablecoin will have the promised reserves. This will in turn increase trust in the financial sector. As a result, the European Commission’s first option would allow for greater crypto-asset adoption.

The second option would be to regulate stablecoins following the definition of the Electronic Money Directive, with the value of stablecoins backed by a single currency that serves as legal tender. This is similar to the Electronic Money Directive’s definition of e-money (European Commission 2019, 8). Stablecoins, like e-money, can function as a means of payment and, when backed by a reserve asset, can function as a credible means of exchange or store of value. However, because the EMD2 was not designed to govern stablecoins, it may not mitigate their risks to consumers.

After considering the two options presented above, the European Commission decided to combine them in the MiCA Regulation to allow the governance of the various functions of stablecoins and the establishment of a comprehensive EU framework to mitigate the risks identified by the FSB (2019).

Interestingly, and although a regulation is clearly required, this approach has also been criticized. ESMA, in particular, noted that broadening regulations for crypto-assets could result in certain trade-offs, such as the risk of legitimizing crypto-assets and encouraging adoption, which would necessitate additional supervisory resources (ESMA 2019, 40). However, since 2018, crypto-assets have seen a significant increase in popularity among retail customers, with institutional investors also expressing an interest in investing in crypto-assets and using DLT to establish innovative solutions. As a result, it can be argued that what is required is regulation that promotes innovation while also preserving financial stability, market integrity and investor protection.

## Proposed MiCA regulation

This section assesses the MiCA Regulation’s scope and legal basis, the gaps it will fill in comparison to current legislation, and its applicability and effects in the financial sector.

### Objectives of the MiCA regulation

The Commission proposed the MiCA Regulation as the first comprehensive regulation directly addressing crypto-assets, with the aim of boosting innovation while preserving financial stability and market integrity and protecting investors from risks (European Commission 2020). MiCA thus regulates a new asset class, crypto-assets, which differs from digital securities such as stocks and bonds. MiCA, in conjunction with existing legislative frameworks, has been drafted to cover the entire crypto-asset ecosystem, leaving no crypto-asset unregulated. It aims to accomplish four major goals.

Its first goal is to provide legal certainty (European Commission 2019, 2). To develop crypto-asset markets in the EU, we need a solid legal framework that clearly defines the rules that apply to all crypto-assets that are not covered by existing financial legislation. The second goal is to establish a legal framework that is both safe and proportionate, promoting innovation and fair competition (European Commission 2019, 2). The third goal is to put adequate levels of consumer and investor protection in place to eliminate the risks that crypto-assets may pose to the internal market. The fourth goal is to ensure financial stability, with the European Commission mentioning stablecoins specifically as having the potential to become widely accepted and cause systemic risks (European Commission 2019, 3). Systemic risks are those that, if not properly managed, could harm the real economy (G7 Working Group on Stablecoins 2019). As a result, the MiCA Regulation seeks to address the potential risks to financial stability and monetary policy posed by crypto-assets, particularly stablecoins.

The proposed MiCA Regulation imposes various requirements on crypto-asset issuers and service providers, including obtaining prior documentation from their NCA. This documentation will serve as an EU Passport for authorization across the single market.^14^ MiCA also creates new sets of EU rules, such as the requirement to create a white paper.^15^

### Scope

The scope of MiCA generally includes all currently unregulated crypto-assets that are not covered by other EU financial legislation, as well as crypto-asset issuers and crypto-asset service providers that provide services in the European Union. MiCA does not cover:

- Financial instruments under Article 4(1) and (15) MiFID II;
- E-money, as defined in Article 2(2) E-Money Directive;
- Deposits as defined in Article 2(1) and (3) Deposit Guarantee Schemes Directive;
- Structured deposits as defined in Article 4(1) and (43) MiFID II;
- Securitization as defined in Article 2(1) Securitisation Regulation (Sygna 2020).^16^

MiCA’s obligations also do not apply to the European Central Bank (ECB), national central banks of Member States, the European Investment Bank, the European Financial Stability Facility, the European Stability Mechanism, and public international organizations.^17^

Although MiCA applies to crypto-asset issuers and crypto-asset service providers (CASPs), depending on the circumstances, various exemptions may apply. Article 3(6) defines issuers as any legal person who offers any type of crypto-asset to the public or seeks admission of such crypto-assets to a crypto-asset trading platform. CASPs are defined in Article 3(8) as any person whose occupation or business is the professional provision of one or more crypto-asset services to third parties.

### Legal basis

The proposed MiCA Regulation is based on Article 114 of the Treaty on the Functioning of the European Union (TFEU), which lays the legal groundwork for establishing an internal market (European Commission 2019, 4). It empowers the EU to enact legislation to harmonize any national laws that may impede the free movement of goods, services, capital, or people, thus contributing to the internal market’s obstruction. Some EU Member States already have crypto-asset regulations in place (Novaković 2021; Inozemtsev 2021).

The MiCA Regulation has been proposed following the subsidiarity principle, which allows the Union to intervene and take action when the objectives of an action cannot be adequately achieved by the Member States on their own (Pavy 2021). This principle is defined in Article 5 of the Treaty on European Union. It ensures that decisions are made as close to citizens as possible, with constant checks to ensure that actions at the EU level are consistent with the opportunities available at the national, regional, or local levels. The MiCA Regulation was proposed in light of this subsidiarity principle, as the national and regional levels were clearly inadequately equipped to achieve the objectives. As a result, the EU took action to regulate crypto-assets because the impact would be much greater at the EU level than at the national level, especially given the need for uniformity among EU Member States.

The proposed MiCA Regulation is also subject to the proportionality principle, which states that the content and form of any EU action should not exceed what is necessary to achieve the Treaty’s objectives. As stated in “Defining the problem and the emergence of the MiCA Regulation” section, the goals of MiCA include achieving legal certainty, fair competition, investor protection, and financial stability. These goals are intended to be met at the EU level because Member States cannot achieve them independently. MiCA ensures proportionality by design, which means distinguishing each type of service and activity based on the associated risks. The Commission stated that the requirements for stablecoins are more stringent because these have been identified as growing at a faster rate, potentially resulting in higher levels of risk. This is consistent with MiCA Recitals 4 and 5, which express a desire to harmonize legislation to avoid regulatory fragmentation.

### Types of tokens

The MiCA Regulation applies to three types of tokens:

1. Asset-referenced tokens;
2. E-money tokens;
3. Other crypto-assets.

All three types are discussed below, and a summary is given in Table 2. This classification does not overlap with the types of crypto-assets discussed in "Types of crypto-assets" section above, primarily concerned with the function of the tokens. Cryptocurrencies can fall into any of these three categories, depending on their worth. Utility tokens are likely other crypto-assets, but if they have an investment component, they may also qualify as asset-referenced tokens. Security tokens are most likely asset-referenced tokens, though they may already qualify as a financial instrument under MiFID (and so not be covered by MiCA).

**Table 2:** Types of tokens in MiCA

Token in MiCA	Relevant articles	Definition	Function
Asset-referenced	Definition 3(1)(3) Title III, arts. 15–42	A type of crypto-asset that purports to maintain a stable value by referring to the value of several fiat currencies that are legal tender, one or several commodities or one or several crypto-assets, or a combination of such assets	Exchange or security
e-money	Definition 3(1)(4) Title IV, arts. 43–52	A type of crypto-asset the main purpose of which is to be used as a means of exchange and that purports to maintain a stable value by referring to the value of a fiat currency that is legal tender	Exchange
Other	Title II, arts. 4–14	Not asset-referenced or e-money	Exchange or utility or security

The first category, asset-referenced tokens, includes crypto-assets that claim to have a stable value by referencing the value of one or more fiat currencies, commodities, crypto-assets, or a combination of such assets.^18^ According to Article 15, no asset-referenced token may be offered in the EU or admitted to trading on a crypto-asset trading platform unless it is offered by an entity established in the EU or an entity authorized per Article 19. Smaller offers (less than EUR 5 million) and offers only to qualified investors are exempt from this authorization. Individuals with significant wealth, according to MiCA, will be able to acquire crypto-assets before ordinary citizens and without being subject to the same regulatory hurdles (Cengiz 2021).^19^ The exemption from authorization also applies to credit institution issuers, such as banks.^20^ Asset-referenced tokens can be significant if classified by the EBA on its own initiative, per Article 39, or at the issuer’s request, in accordance with Article 40. When determining significance, the asset-referenced token is evaluated against the following criteria: the size of the promoters’ customer base; the value of the asset-referenced tokens or market capitalization; the size of the assets reserves; the significance of cross-border activity, including the use of cross-border payments; and the level of interconnectedness with the financial system.^21^

Significant asset-referenced token issuers must meet additional criteria, such as a remuneration policy, interoperability requirements, a liquidity management policy, and higher own funds requirements. Furthermore, supervision will be delegated to and governed by the EBA.^22^ Diem (Diem Association 2022), which is assumed to be at the heart of the MiCA proposal, would most likely qualify as a significant asset-referenced token and thus be well-regulated and supervised under MiCA.

The second category, e-money tokens, includes crypto-assets used as a means of exchange that maintain a stable value by referring to the value of a single legal tender fiat currency. As a result, these tokens differ from asset-referenced tokens in that they refer to the value of a single fiat currency, whereas asset-referenced tokens refer to the value of one or more fiat currencies, commodities, or crypto-assets. The same criteria can be used to identify significant e-money tokens as for asset-referenced tokens. The EBA has the authority to classify an e-money token as significant on its own initiative, in accordance with Article 50, or at the request of an issuer, under Article 51. Issuers of significant e-money tokens are subject to additional requirements, such as rules on reserve asset custody, rules on reserve asset investment, higher own funds requirements, and an orderly wind-down plan.^23^

The third category, other crypto-assets, covers all crypto-assets other than asset-referenced and e-money tokens. This category will most likely include utility tokens, which allow access to goods or services and cryptocurrencies. However, because the current negative definition is so broad, this latter aspect is currently unknown. This is one of the points of criticism of the proposed MiCA Regulation.

### Obligations for crypto-asset issuers and CASPs

The MiCA proposal imposes various obligations on crypto-asset issuers and CASPs for each type of token.

#### Other crypto-assets

All other crypto-assets are subject to the general obligation to comply with Article 4, which states that no issuer of crypto-assets (unless these assets are asset-referenced or e-money tokens) may make an offer to the public in the EU or seek to trade crypto-assets on a trading platform unless the issuer is established as a legal entity, has drafted a crypto-asset white paper, has notified this crypto-asset white paper to an NCA in its home Member State and complies with the requirements laid down in Article 13.

Issuers of these other crypto-assets must therefore notify the NCA of the white paper, though approval is not required.^24^ After publishing its crypto-asset white paper, the issuer is now able to offer its crypto-assets throughout the EU. This is because the EU Passport^25^ allows issuers to issue crypto-assets anywhere in the EU without requiring additional approval, because an authenticated EU Passport is valid throughout the EU.

The following situations are exempt from the requirement to draft, notify, and publish a white paper:

- Free offers;
- Crypto-assets for the maintenance of DLT;
- Small offers (i.e., to fewer than 150 people per Member State);
- Small offers (i.e., involving total consideration of less than 1 EUR million);
- Offers solely to qualified investors;
- Crypto-assets that are unique and not fungible;
- Crypto-assets offered to the public or admitted to trading before MiCA enters into force.^26^

Although these exemptions may benefit smaller fintech start-ups and crypto exchanges, the threshold for small offers is relatively high, which may be interpreted as discouraging smaller fintechs from growing, given that growth will result in additional costly and time-consuming obligations. Qualified investors are defined in Article 2(e) Prospectus Regulation as persons or entities listed in points (1) to (4) of Section I, Annex II, of MiFID II/MiFIR and persons and entities treated as professional clients in Section II of that Annex. These categories therefore exempt offerings to various players, including larger players like investment firms and institutional investors, as well as large undertakings that meet two of the size requirements specified in Annex II (2) MiFID II/MiFIR. As a result, offerings of other crypto-assets to qualified investors may have an unfair advantage.

#### Asset-referenced tokens

Asset-referenced tokens must comply with Article 15, which states that such tokens can only be offered in the EU or admitted to trading if offered by a legal entity established in the EU and accompanied by a white paper issued and approved by the NCA.

An application to issue asset-referenced tokens must be authorized in accordance with Article 19. An exemption from authorization applies for:

- Small offers (less than EUR 5 million in the EU);
- Offers addressed solely to qualified investors and that can be held only by qualified investors;
- Issuers that are credit institutions.

#### E-money tokens

According to Article 43, issuers of e-money tokens must be a legal entity established in the EU and licensed as a credit institution or an e-money institution, and must publish a white paper following Article 46 and Annex III.

These criteria are waived for small offers (less than EUR 5 million) or if qualified investors only hold the e-money tokens. This highlights the unfair conditions explored in "Other crypto-assets" section, which exist for both asset-referenced and e-money tokens.

#### CASPs

The obligations imposed on CASPs governed by the MiCA are very similar to those imposed on financial services governed by financial service regulations. A CASP typically performs one or more of the following activities:

- Operation of a crypto-asset trading platform;
- Exchange of crypto-assets for legal tender fiat currency;
- Exchange of crypto-assets for other crypto-assets;
- Execution of orders for crypto-assets on behalf of third parties;
- Placing of crypto-assets;
- Reception and transmission of orders for crypto-assets on behalf of third parties;
- Providing advice on crypto-assets;
- Custody and administration of crypto-assets on behalf of third parties.^27^

Under Article 53 MiCA, any CASP wishing to operate has to be a legal person with a registered office in the EU and authorized by the competent authorities. An exemption applies for existing credit institutions and MiFID II investment firms. Therefore, the obligations imposed on CASPs are similar to those imposed on traditional financial services by regulatory provisions. These regulatory requirements pertain to initial capital reserves, IT infrastructure security, corporate governance structure, and the suitability of the management board. In Germany, for example, where licenses for certain crypto-related financial services already exist,^28^ simplified authorization procedures will be implemented to upgrade those licenses.^29^ This could give EU Member States that are already pioneers in the crypto space a competitive advantage, as they may be able to obtain new licenses more quickly. Once approved, the service can be approved in the rest of the EU via passporting.^30^

## Impact of MiCA on crypto-asset adoption

This section explores the impact that MiCA may have on crypto-asset adoption in the financial services sector, as well as the benefits of MiCA and the concerns raised by various stakeholders. Finally, amendments to the current proposal will be considered to examine whether MiCA can indeed facilitate crypto-asset adoption in the financial sector.

### Benefits for crypto-asset adoption

As discussed in "Objectives of the MiCA regulation" section, the MiCA Regulation was designed to provide legal certainty by establishing a legal framework that encourages innovation and fair competition while also protecting investors and consumers. Several factors clearly contributed to the emergence of MiCA, including a lack of certainty about how existing EU rules apply to crypto-assets, the lack of crypto-asset rules at the EU level, and diverging national crypto-asset rules (Dentons 2020). MiCA is thus intended to reduce regulatory barriers to the use of crypto-assets, to reduce the risks of fraud and consumer and investor protection, and to reduce the risks of market integrity, market fragmentation, and the risks of not achieving a level playing field and financial stability, while also addressing monetary policy concerns (Dentons 2020).

In general, MiCA will indeed create legal certainty by establishing a uniform legal framework that is directly applicable in the Member States. Institutions such as the ECB have anticipated and welcomed a regulation for crypto-assets. MiCA clearly states, in Article 2 that it applies to anyone offering crypto-assets or providing crypto-asset services in the EU. Article 2(2) of the MiCA Regulation ensures that the regulation only applies to crypto-assets that are currently unregulated and fall outside the scope of existing financial services legislation. Therefore, anyone issuing a financial instrument covered by MiFID II/MiFIR will remain subject to that legislation.

As discussed in "Legal basis" section, any party issuing tokens under MiCA must be a legal entity. This means that serious tokens will no longer be issued anonymously or by amateurs. The requirement for a legal entity ensures that an accountable entity can be found and sued if necessary. Due to the legal certainty provided by this requirement, consumers and investors may have more trust in crypto-assets, contributing to crypto-asset adoption.

However, a legal framework does not meet the objectives that have been set. The context addressed by a regulation must also be considered, with compliance and enforcement being especially important issues to consider. Indeed, several institutions and individuals have expressed concerns about the MiCA Regulation’s applicability and appropriateness for the crypto-asset ecosystem.

### Key concerns and considerations for crypto-asset adoption

The proposed MiCA Regulation resulted in various opinions and suggestions being published. This paper considers the views of the ECB, the International Association for Trusted Blockchain Applications (INATBA), the European Economic and Social Committee (EESC), and rapporteurs. The ECB, a key stakeholder, issued its opinion on the MiCA Regulation in accordance with Articles 127(4) and 282 (5) TFEU (European Central Bank 2021). The ECB can issue an opinion because the proposed regulation contains provisions within its competence, such as its responsibility for monetary policy, the promotion of the smooth operation of payment systems, prudential supervision of credit institutions, and contributing to the smooth implementation of policies pursued by competent authorities relating to financial market stability. Another influential opinion came from INATBA, a platform of 105 organizations representing the entire DLT ecosystem that is hosted at the European Commission headquarters. INATBA’s proposals are thus a direct reflection of the views of consumers and investors investing in crypto-assets, and they are crucial. The EESC is an EU advisory body comprised of representatives from labor and employer organizations, as well as other interest groups, whose mission is to ensure that EU law is geared toward economic and social conditions. The EESC’s opinion on the MiCA Regulation is highly valued by the crypto-asset ecosystem because it represents workers, employers, and other interest groups. Lastly, the rapporteurs are European Parliament members who are in charge of handling the legislative proposal for the MiCA Regulation, both procedurally and substantively (European Parliament 2020). Their opinion is therefore highly influential and significant in the event that the current proposal is amended.

The key concerns that will be examined here are the concerns that:

1. The broadness of the definitions in MiCA will create a lack of legal certainty;
2. An uneven playing field will cause unfair competition;
3. A lack of supervisory requirements will limit financial stability;
4. Stringent requirements will stifle innovation.

The first concern is the breadth of crypto-asset definitions. According to the ECB, the definition of a crypto-asset in MiCA is very broad and catch-all, and thus not very clear (European Central Bank 2021, § 1.4). Changes are specifically requested regarding stablecoin supervision (in MiCA terminology: asset-referenced tokens). The ECB has also advocated for additional safeguards for asset-referenced tokens, such as prudential and liquidity requirements for token issuers that are proportionate to the risks these tokens may pose to financial stability. This is also supported by INATBA, which is concerned that the broadness of the definitions will make consistent application across Member States difficult. INATBA specifically refers to crypto-assets and utility tokens that may inadvertently bring into scope projects and products that are not intended to be used for investment or finance purposes (INATBA 2021). Furthermore, the definitions do not go into detail about hybrid tokens, which contain elements of a security token and may perform different functions after issuance. Another issue to consider is how derivatives based on crypto-assets should be classified: as a financial instrument (and thus not covered by MiCA) or as an asset-referenced token (and thus covered by MiCA)? These opinions demonstrate that the broadness and broad scope of MiCA definitions contribute significantly to legal uncertainty (Zetzsche et al. 2021), thus defeating the legislation’s purpose.

Another source of concern is the legal uncertainty surrounding MiCA’s position concerning existing legislative frameworks (Lannoo 2021). The ECB has requested that the scope of the definitions of crypto-assets subject to MiCA Regulation, on the one hand, and those subject to the MiFID II/MiFIR framework, on the other, be clarified (Dentons 2021). Regulatory frameworks should not be in conflict with one another, but rather should be aligned to avoid regulatory uncertainty, excessive compliance costs and burdens on operators, and potentially limiting innovation (European Economic and Social Committee 2020). The ECB observes that the potential interaction between MiCA and PSD2 will require further consideration by the co-legislators (the European Council and Parliament), as will the question of whether CASPs contracting with a payee to accept crypto-assets other than e-money tokens must meet the same consumer protection, security, and operational resilience requirements as regulated PSD2 payment service providers. It will also be necessary to clarify whether such activities should be interpreted as payment transaction acquisition, as defined by PSD2 (European Central Bank 2021 § 2.2.4).

Concerned about the alignment of existing legal frameworks, rapporteur Berger stated that crypto-assets should be subject to the same rules as more traditional financial instruments, particularly in terms of AML and CTF requirements (European Parliament 2020, proposed amendment 11; Bogart 2021). Berger also suggested that the distinction between an asset under MiCA and a financial instrument under the MiFID II/MiFIR framework be clarified. This should adhere to the same risks and rules principle (European Commission 2020), which is also intended to ensure technological neutrality.

It can be difficult to ensure that a regulation clearly defines the categories of objects to which it applies. Much depends on the perspective chosen and whether the definition is based on a technical point of view (analog/digital; recorded in a central database or on a decentralized ledger), an economic point of view (transferable; instrument of payment; investment objective; what it exactly stands for), or the interests to be protected (no money laundering; no terrorist financing; financial stability; monetary sovereignty). The legislative acts that preceded MiCA, including the gaps intended to be filled by MiCA, were established from various perspectives. As a result, the definitions used in this legislation for the objects covered are not always mutually consistent or compatible. The result is a patchwork quilt of concepts such as virtual assets, crypto-assets, virtual currencies, cryptocurrencies, electronic money, financial instruments, payment instruments, and transferable securities, with the question of how these definitions relate to one another still unanswered. Then, initiatives that emerge in the market do not always appear in a clearly labeled category, and may combine aspects of existing categories to form new hybrids. Trying to regulate in a rapidly evolving field like this is analogous to shooting at a moving target.

So, what does this mean for anyone planning to launch a new crypto-asset or provide crypto-asset-related services? They must decide which regulations to take into consideration. Is their crypto-asset classified as a financial instrument under MiFID? Is it electronic money or virtual currency? Which rules are in effect? As a result of the resulting uncertainty, the question of whether the goal of legal certainty will be met can undoubtedly be raised (Baker and Werbach 2021, p. 172).

The second source of concern is the proposed regulation’s potential to create an uneven playing field. Giving some actors an unfair competitive advantage would counter MiCA’s goal of encouraging innovation and fair competition. Regarding technology neutrality, INATBA has stated that the proposed regulation fails in that it limits the capital raised for DLT projects to a maximum of EUR 1 million in 12 months (INATBA 2021, issue 5). Projects that do not use DLT will gain an unfair advantage because, according to the Prospectus Regulation, they will be able to raise up to EUR 8 million in capital within 12 months. These concerns clearly demonstrate that MiCA does not make adequate provisions to ensure technological neutrality. This may have ramifications for the concept of a level playing field in the crypto-asset ecosystem (Lannoo 2021).

Furthermore, credit institutions are exempt from the requirement to seek authorization under MiCA.^31^ Because banks and investment firms do not need CASP authorization, they can provide crypto-asset services much more easily than firms that must first be authorized. The Commission did not believe that additional authorization was required because banks and investment firms are already subject to stringent supervisory requirements and regulations. On the other hand, small companies, start-ups, crypto exchanges, and fintechs must have a registered office in a Member State, complete the legal entity documentation, and issue a white paper. This disadvantages smaller firms because they may lack the financial resources to become a CASP. Furthermore, only banks and e-money institutions are permitted to issue e-money tokens. Preventing other players from entering the e-money token market in this manner gives those credit institutions a competitive advantage (GFIA Info 2021).

The third source of concern is the absence of supervisory requirements in MiCA (Zetzsche et al. 2021). The ECB notes that greater ECB oversight is required for financial stability and prudential supervision, as well as clarification of how ECB supervision interacts with oversight provided by other European Supervisory Authorities, such as the EBA, as well as dual supervision by NCAs or, in the context of the Banking Union, tri-party supervision (Dentons 2021, § 3.1.4). According to the ECB, a clearer distinction is required between the EBA’s responsibilities (which are given primary oversight powers under MiCA) and the ECB’s existing powers as the head of the Banking Union’s Single Supervisory Mechanism. The ECB can currently only provide non-binding opinions on crypto-asset white papers, limiting its ability to supervise the crypto-asset ecosystem. Berger believes the ECB should have supervisory authority over significant crypto-assets and asset-referenced tokens (European Parliament 2020, proposed amendment 10). Shadow rapporteurs agree with Berger that the ECB should have a greater supervisory role, and they also support a stricter approach to significant asset-referenced and e-money tokens (Bogart 2021).

The fourth and final point raised here is the number of stringent requirements included in the MiCA proposal. To begin, INATBA observes that certain proposed requirements could be problematic for decentralized projects, stifling innovation in Europe. DeFi (decentralized finance) projects typically lack central control and may thus be out of scope. Due to the lack of a central party that can be linked to a territory, such projects cannot be attributed to a single jurisdiction. As a result, it may be difficult for them to provide the necessary documentation for qualification and authorization as a CASP. The question that arises is whether and, if so, how DeFi projects could even have a legally accountable entity (Maia and Vieira dos Santos 2021). This raises concerns about the DeFi ecosystem’s innovation (INATBA 2021, issue 2).

Furthermore, the current framework lacks transitional provisions, which means that issuers of e-money tokens and asset-referenced tokens must be authorized as soon as the legislation is enacted. As a result, the registration and authorization process must begin ahead of time and be completed before MiCA takes effect. This could put such issuers at an unfair disadvantage because they will be penalized and unable to take advantage of the 18-month transitional period applicable to other crypto-assets. These stringent requirements demonstrate that the current proposal may stifle rather than promote innovation (INATBA 2021, issue 4).

As a result, while MiCA will provide opportunities for significant benefits through a harmonized framework designed to provide legal certainty, the concerns raised by various institutions and individuals point to potential negative consequences for crypto-asset adoption in financial services. These concerns may affect all parties in the financial services sector, though smaller players will undoubtedly be more impacted than larger competitors (Ferrarini and Giudici 2021).

### Solutions for accelerated crypto-asset adoption

As the first EU-wide regulation governing out of scope crypto-assets, MiCA is the result of a significant shift in thinking, with regulators becoming aware of the significance and potential for increased adoption of crypto-assets. Although various institutions and individuals have expressed reservations about the MiCA proposal in its current form, it has also been lauded as an important step toward crypto-asset adoption in the EU, and potential solutions to these reservations have been proposed.

First, in terms of definition broadness, INATBA has proposed amending the definitions of crypto-assets to make them more activity based, such as tokens with specific payment functionalities (INATBA 2021, issue 1). It is also critical that the definitions be binding rules rather than mere guidelines. The EESC believes that more detailed specifications for the various subcategories of crypto-assets, particularly the definition of other crypto-assets, can help to achieve legal clarity (European Economic and Social Committee 2020). MiCA should provide alternative definitions for DLT, e-money, and utility tokens, according to the Global Digital Finance Working Group, in order to achieve greater clarity (Global Digital Finance 2021). Furthermore, greater alignment with existing EU financial services legislation, particularly MiFID II/MiFIR and PSD2, is required. If the definitions are not appropriately amended, the lack of legal certainty may cause crypto-asset adoption in the EU to slow, as investors and consumers alike may lose trust in these assets, and institutions may be hesitant to offer DLT-based solutions.

Second, in order to avoid the uneven playing field that will be created by exempting credit institutions from the requirement to seek authorization under MiCA, INATBA proposes that this exemption be removed, so that credit institutions must meet the same requirements as other issuers (INATBA 2021, issue 3). However, it is questionable whether imposing the same administrative burdens on already heavily regulated and supervised credit institutions solely to create a level playing field is wise or efficient. Even if the additional requirements increase the costs of entering the crypto-asset ecosystem for smaller firms and start-ups, these costs may be justified in the long run because these entities can provide crypto-asset services safely and securely. Furthermore, the passport license that will be issued upon approval will be costly in the short term, but will pay off in the long run by allowing CASPs to provide their services in all Member States.

Third, in response to the lack of supervisory oversight, Berger proposes that the ECB be designated as the governing authority with greater supervisory authority (European Parliament 2020, proposed amendment 11; Bogart 2021). Applications for authorization to issue asset-referenced and e-money tokens must currently be approved by the NCA before being sent to the EBA. Currently, the ECB can only provide non-binding opinions on these white papers. This should be changed to binding opinions to give the ECB more power.

Fourth, MiCA imposes some onerous requirements that may stifle innovation. Concerning the issue of DeFi projects falling outside the scope of MiCA, INATBA suggests that such projects rely on an alternative structure, with these projects represented by a foundation (INATBA 2021, issue 2). The foundation would act as the legal entity and thus the point of contact for regulators and supervisors, allowing DeFi projects to be classified as MiCA-compliant. Concerning the lack of transitional arrangements for e-money and asset-referenced tokens, such arrangements is proposed to be harmonized across all token categories to create a level playing field. Berger proposes stricter requirements, particularly for CASPs. He wants CASPs to have exit strategies in place, and he believes that providers who transfer crypto-assets for payment purposes should be able to track all transfers within the EEA.^32^ The feedback on the stringent requirements indicates that, in general, strict rules will be required to ensure a legal framework capable of regulating crypto-assets. As a result, while the MiCA Regulation imposes new requirements, they should not prevent crypto-asset adoption. However, the effects of the MiCA framework on crypto-asset adoption will only be felt and measured once the regulation is implemented.

## Conclusion

Although some may remain skeptical of crypto-assets, they appear to be here to stay. This study emphasizes the importance of effective regulation. It was decided to explore the effects of the EU MiCA Regulation on crypto-asset adoption in the financial services sector on this occasion because this sector has been identified as having tremendous potential in the crypto space, particularly among crypto exchanges and traditional finance market operators. The study explained the DLT properties that form the foundations of crypto-assets’ decentralized nature, highlighting the risks of decentralization.

MiCA has been proposed as part of the EU’s Digital Finance Package to ensure that crypto-assets, which are currently unregulated, are governed by a consistent framework. Existing legislation relevant to the MiCA framework includes MiFID II/MiFIR, AMLD5, EMD2, and the PSD2, all of which cover different aspects of the financial services sector and will continue to apply. The emergence of the global stablecoin project Diem, risks identified by European Supervisory Authorities, a study conducted by the European Commission, and gaps in the existing legislative framework all contributed to the need for MiCA. A particular need highlighted was the need to regulate stablecoins, which led to the proposed regulation’s stringent token categories of asset-referenced tokens and e-money tokens, with the goal of ensuring legal certainty, a legal framework to support innovation and fair competition, appropriate levels of consumer and investor protection, and financial stability.

This study aimed to determine whether the proposed MiCA Regulation can be expected to create legal certainty and thus allow for the anticipated increased adoption of crypto-assets in financial services.

The paper’s findings indicate that creating a harmonized legal framework may result in greater legal certainty, whereas the requirement to be a legal entity and issue a white paper may result in greater accountability for crypto-asset issuers and providers. However, our examination of the MiCA framework revealed that the consequences of the various concerns raised may outweigh the benefits of the regulation. These concerns include the broadness of crypto-asset definitions; MiCA’s ambiguous position in relation to existing legislation; an uneven playing field that gives larger players, particularly credit institutions and qualified investors, a competitive advantage; a lack of supervisory oversight, particularly by the ECB; and too strict requirements that fail to provide scope for DeFi projects or for transitional arrangements.

The paper explored various solutions that could help MiCA achieve its goal of enabling regulated crypto-asset adoption. These include amending and clarifying MiCA definitions, removing credit institution exemptions, arranging for increased ECB supervision, and introducing additional requirements, such as provisions for transitional arrangements and arrangements more closely aligned with conditions for other tokens and existing legal frameworks.

We conclude that the MiCA Regulation, as currently proposed, will most likely not facilitate accelerated adoption of crypto-assets in the EU financial services sector, at least not sufficiently or as intended. However, it is hoped that implementing the aforementioned solutions will aid the MiCA Regulation in increasing crypto-asset adoption, given that, in the long run, and assuming the proposal is amended, it has the potential to regulate an ecosystem of crypto-assets that are currently unregulated satisfactorily. Therefore, let us hope for the realization of the Commission’s Digital Finance Package dreams and that European citizens and businesses alike will eventually be able to profit safely from all of the benefits that crypto-assets can provide.

## Data Availability

Not applicable.

## Abbreviations

AML: Anti-money laundering
AMLD5: 5Th anti-money laundering directive
BEUC: Bureau Européen des Unions de Consommateurs
CASP: Crypto-asset service provider
CFT: Counter-terrorism financing
DeFi: Decentralised finance
DLT: Distributed ledger technology
EBA: European Banking Authority
ECB: European Central Bank
ECON: European Parliaments Committee on Economic and Monetary Affairs
EEA: European Economic Area
EESC: European Economic and Social Committee
EFSF: European Financial Stability Facility
EIB: European Investment Bank
EIOPA: European Insurance and Occupational Pensions Authority
EMD2: Second Electronic Money Directive
ESMA: European Securities and Markets Authority
FATF: Financial Action Task Force
FSB: Financial Stability Board
GDP: Gross domestic product
GDPR: General Data Protection Regulation
ICO: Initial coin offering
INATBA: International Association for Trusted Blockchain Applications
KYC: Know Your Customer
MiCA: Markets in Crypto-Assets
MiFID II: Markets in Financial Instruments Directive Framework II
MiFIR: Markets in Financial Instruments Regulation
OECD: Organisation for Economic Co-operation and Development
PSD2: Second Payment Services Directive
SMSG ESMA: Securities and Markets Stakeholder Group of ESMA
STO: Security Token Offering
TEU: Treaty on European Union
TFEU: Treaty on the Functioning of the European Union
VASP: Virtual asset service providers

## Footnotes

1 Proposal for a Regulation of the European Parliament and of the Council on Markets in Crypto-assets, and amending Directive (EU) 2019/1937, COM/2020/593 final.

2 See for example the following Youtube tutorials: *How to create your own token on Solana*: https://youtu.be/1cn-HnG_yns; *Create a token on Binance Smart Chain*: https://youtu.be/Q_wK6N9GtS8; *Create an ERC20 token on Ethereum*: https://youtu.be/ZLFiGHIxS1c; *Build a Crypto token with no coding*: https://youtu.be/2F9n_f0p5gI, or even *How to make your own cryptocurrency scam*: https://youtu.be/BGul38vjlGk, and many more. There are even token generators, such as https://vittominacori.github.io/erc20-generator/.

3 *Securities and Exchange Commission v. W.J. Howey Co.*, 328 U.S. 293 (1946).

4 Council of the European Union, Interinstitutional File: 2020/0265 (COD), Brussels, 5 October 2022, https://data.consilium.europa.eu/doc/document/ST-13198-2022-INIT/en/pdf.

5 Council Directive 2014/65/EC of 15 May 2014 on markets in financial instruments and amending Directive 2002/92/EC and Directive 2011/61/EU [2014] OJ L173/349.

6 Council Regulation (EC) 600/2014 on markets in financial instruments and amending Regulation OJ L648/2012.

7 Council Directive 2014/65/EC of 15 May 2014 on markets in financial instruments and amending Directive 2002/92/EC and Directive 2011/61/EU [2014] OJ L173/349.

8 Council Directive 2018/843/EC of 30 May 2018 amending Directive (EU) 2015/849 on the prevention of the use of the financial system for the purposes of money laundering or terrorist financing, and amending Directives 2009/138/EC and 2013/36/EU [2018] OJ L156/43.

9 A custodian wallet provider is an entity that provides services to safeguard cryptographic keys on its customers behalf.

10 Directive 2009/110/EC of 16 September 2009 on the taking up, pursuit and prudential supervision of the business of electronic money institutions amending Directives 2005/60/EC and 2006/48/EC and repealing Directive 2000/46/EC [2009] OJ L337/35.

11 Council Directive 2015/2366/EC of 25 November 2015 on payment services in the internal market, amending Directives 2002/65/EC, 2009/110/EC and 2013/36/EU and Regulation (EU) No 1093/2010, and repealing Directive 2007/64/EC [2015] L337/35.

12 Art. 126 (3) Proposed MiCA Regulation.

13 Communication from the Commission to the European Parliament, the Council, the European Economic and Social Committee and the Committee of the Regions on a Digital Finance Strategy for the EU, COM/2020/591 final.

14 Artt. 15 and 41 Proposed MiCA Regulation.

15 Art. 5 Proposed MiCA Regulation.

16 Art. 2(2) Proposed MiCA Regulation.

17 Art. 2(3) Proposed MiCA Regulation.

18 Art. 3(1)(3) Proposed MiCA Regulation.

19 Art. 3(1)(20) Proposed MiCA Regulation.

20 Art. 5(4) Proposed MiCA Regulation.

21 Art. 39(1) Proposed MiCA Regulation.

22 Art. 41 Proposed MiCA Regulation.

23 Art. 52 Proposed MiCA Regulation.

24 Art. 7(1) Proposed MiCA Regulation.

25 Art. 10 Proposed MiCA Regulation.

26 Art. 4(2) Proposed MiCA Regulation.

27 Artt. 59-73 Proposed MiCA Regulation.

28 See, for example, Article 32 of the German Credit Act [*Kreditwesengesetz*, KWG].

29 Art. 54(3) Proposed MiCA Regulation.

30 Art. 53(3) Proposed MiCA Regulation.

31 Article 2(3), (4) and (5) Proposed MiCA Regulation.

32 Identifying the steps and resources required to wind-down a business, especially where resources are limited.
